# Polysaccharide Matrices for the Encapsulation of Tetrahydrocurcumin—Potential Application as Biopesticide against *Fusarium graminearum*

**DOI:** 10.3390/molecules26133873

**Published:** 2021-06-24

**Authors:** Anne Loron, Vesta Navikaitė-Šnipaitienė, Deimantė Rosliuk, Ramunė Rutkaitė, Christian Gardrat, Véronique Coma

**Affiliations:** 1 Laboratoire de Chimie des Polymères Organiques, Université de Bordeaux, CNRS, Bordeaux INP, UMR 5629, 16 Avenue Pey-Berland, F-33600 Pessac, France; anne.Loron@enscbp.fr (A.L.); christian.gardrat@u-bordeaux.fr (C.G.); 2 Department of Polymer Chemistry and Technology, Kaunas University of Technology, Radvilenu Rd. 19, LT-50254 Kaunas, Lithuania; vesta.navikaite@ktu.lt (V.N.-Š.); deimante.simanaviciute@ktu.lt (D.R.); ramune.rutkaite@ktu.lt (R.R.)

**Keywords:** OSA-starch, chitosan, tetrahydrocurcumin, freeze-drying, spray-drying

## Abstract

Cereals are subject to contamination by pathogenic fungi, which damage grains and threaten public health with their mycotoxins. *Fusarium graminearum* and its mycotoxins, trichothecenes B (TCTBs), are especially targeted in this study. Recently, the increased public and political awareness concerning environmental issues tends to limit the use of traditional fungicides against these pathogens in favor of eco-friendlier alternatives. This study focuses on the development of biofungicides based on the encapsulation of a curcumin derivative, tetrahydrocurcumin (THC), in polysaccharide matrices. Starch octenylsuccinate (OSA-starch) and chitosan have been chosen since they are generally recognized as safe. THC has been successfully trapped into particles obtained through a spray-drying or freeze-drying processes. The particles present different properties, as revealed by visual observations and scanning electron microscopy. They are also different in terms of the amount and the release of encapsulated THC. Although freeze-dried OSA-starch has better trapped THC, it seems less able to protect the phenolic compound than spray-dried particles. Chitosan particles, both spray-dried and lyophilized, have shown promising antifungal properties. The IC_50_ of THC-loaded spray-dried chitosan particles is as low as 0.6 ± 0.3 g/L. These particles have also significantly decreased the accumulation of TCTBs by 39%.

## 1. Introduction

*Fusarium graminearum* has been classed as the fourth most important fungal plant pathogen regarding scientific and economic criteria [1]. This species is responsible for severe wheat, maize or barley diseases. The presence of rotten parts of plant or damaged kernels can provoke up to 50% of yield loss [2], leading to unpredictable economic losses. Fungal contamination is often associated with the presence of mycotoxins, which are toxic secondary metabolites accumulated by the fungi. These mycotoxins represent a major threat for both human and animal health. *F. graminearum* can specifically produce 4-deoxynivalenol (DON), which is a worldwide-regulated toxin, and some derivatives of DON, all DONs belonging to the trichothecenes B (TCTBs) family. Current strategies to tackle fungal pathogens mostly rely on synthetic fungicides, some of them being endocrine disruptors [3,4,5], being toxic for the environment [6] or inducing the apparition of resistant strains [7].

Our strategy is based on the use of non-toxic, bio-degradable and renewable compounds for the development of antifungal preparations as alternatives to synthetic ones. Tetrahydrocurcumin (THC) is a curcumin derivative, which has already demonstrated promising antifungal and antimycotoxigenic effects against *Fusarium proliferatum*, and has been hardly studied in the literature [8]. Similar to other phenolic compounds, THC also exhibits strong radical scavenging activities [9]. THC has been encapsulated in either modified starch or chitosan matrices, because it is scarcely soluble in aqueous medium and is photosensitive.

OSA-starch is a commercial, easily available and food-approved modified starch derivative. The grafted OSA chains give the starch emulsifying properties. The lipophilic chains are pointing towards the oil phase, whereas the starch units and the charges are in the water phase and repel each other. It has been proven that OSA-starch was at the surface of oil droplets in emulsions [10]. The other employed polysaccharide is chitosan, which is a chitin derivative, obtained from the degradation of crustacean or insect exoskeletons or fungi [11,12]. Chitosan is used in many applications, such as biomedical engineering [13,14,15], drug delivery [16,17,18], packaging [19,20,21,22,23], food preservation [24,25,26,27,28,29,30] or farming improvement [31,32,33,34,35]. Chitosan is a biocompatible polymer and is generally recognized as safe. The association of THC and chitosan could bring increase the efficiency against the fungi and is, therefore, an innovative way to tackle fungal contamination.

Spray-drying and freeze-drying techniques are two common processes to obtain particles loaded with phenolic compounds. THC has already been encapsulated in chitosan nanoparticles [36,37]. Curcumin, the phenolic compound from which THC is derived, has already been trapped into chitosan/Tween 20 spray-dried particles [38], chitosan/Tween 80 spray-dried particles [39] or spray-dried chitosan particles stabilized with the ionic tripolyphosphate linker [40,41]. Curcumin have also recently been loaded in OSA-starch freeze-dried particles [42]. In these examples, the encapsulation of actives has enabled a long-lasting release and a better efficiency.

The feasibility of OSA-starch and chitosan particles with the studied systems has been evaluated with, as a first step, the optimization of the emulsion composition and, as the second step, the study of the influence of the drying process. A brief analysis on THC potential to tackle different strains of *F. graminearum* was then conducted. THC-loaded particles have been prepared and studied. Physicochemical characterizations of those particles by SEM, thermal analysis and release studies have been performed. Finally, the antifungal potential of THC-loaded particles has been assessed on the growth and the TCTB production of a model strain of *F. graminearum*.

## 2. Results

### 2.1. Synthesis of Polysaccharide Particles

#### 2.1.1. Preparation of Emulsions

Aqueous emulsions were made from rapeseed oil and a polysaccharide matrix. In the first case, modified starch was used as matrix and emulsifier, and in the second case, chitosan was used, with Tween 80 as an emulsifier. The parameters (mixing time, speed, ratios) have been optimized to minimize the droplets’ size, as smaller droplets lead to more stable emulsions.

- With OSA-starch

**Figure 1:**
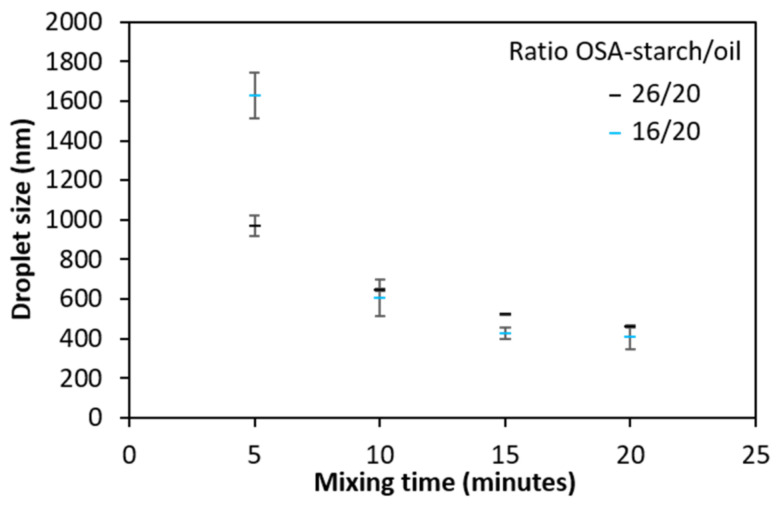
Evolution of the oil droplet size as a function of mixing time for a homogenization speed of 13,000 rpm at two different ratios. Error bars correspond to the standard deviation of measurements in duplicate.

As shown in Figure 1, the longer the time of homogenization, the smaller the size of the droplets. As the size seemed to reach a plateau, a mixing duration of 20 min for the preparation of the emulsions was chosen. For handling reasons, the speed was decreased from 13,000 rpm to 10,000 rpm for the rest of the study.

**Figure 2:**
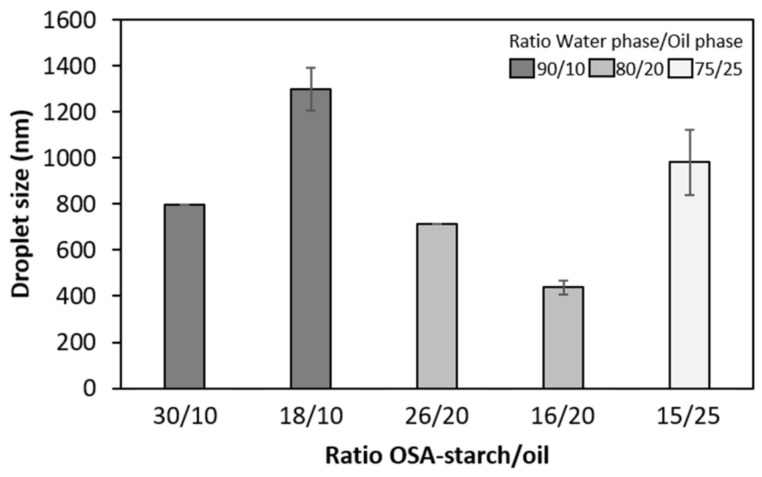
Evolution of the oil droplet size as a function of the OSA-starch/oil ratio for a homogenization speed of 10,000 rpm. Error bars correspond to the standard deviation of measurements in duplicate.

The smallest droplets were obtained with an OSA-starch/oil ratio of 16/20, as can be seen in Figure 2.

As a result, a water phase/oil phase ratio of 80/20 and a OSA-starch/oil ratio of 16/20, with a speed of 10,000 rpm, were chosen for the preparation of the particles in this study. These conditions led to droplets of 438 ± 31 nm diameter, with a polydispersity index of 0.23 ± 0.04.

- With chitosan

**Figure 3:**
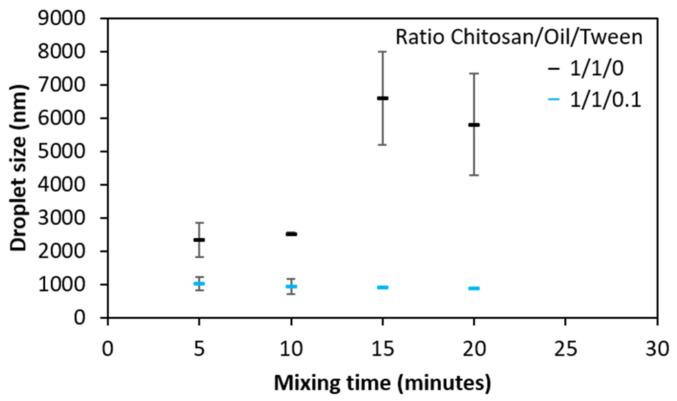
Evolution of the oil droplet size as a function of mixing time for a homogenization speed of 10,000 rpm, with or without Tween 80. Error bars correspond to the standard deviation of measurements in duplicate.

Chitosan emulsions were prepared from an acidic chitosan solution at 1% (*w*/*v*) and Tween 80 as an emulsifier. Without Tween 80, emulsions were not stable, as can be seen in Figure 3, with large droplets.

#### 2.1.2. Drying of Particles

**Figure 4:**
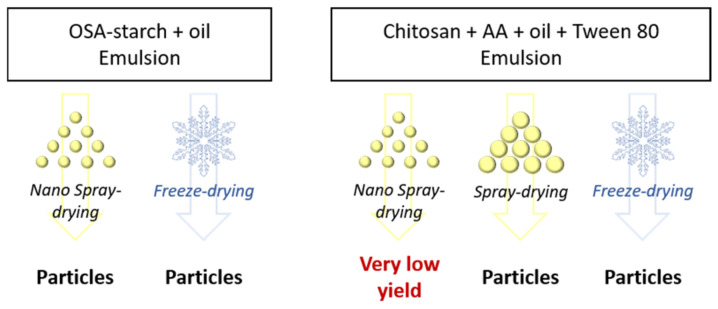
Overview of the techniques to prepare particles.

Emulsions of modified starch or chitosan were dried using two different techniques, quickly after their preparation, as summarized in Figure 4.

- By spray-drying

**Figure 5:**
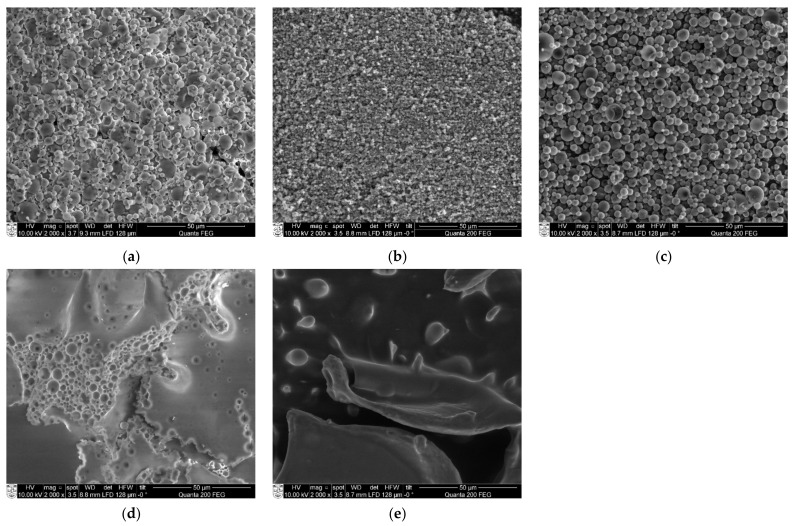
SEM views (2000×) of (**a**) nano spray-dried OSA-starch particles; (**b**) nano spray-dried chitosan particles; (**c**) spray-dried chitosan particles; (**d**) freeze-dried OSA-starch particles; (**e**) freeze-dried chitosan particles.

OSA-starch and chitosan spray-dried particles were analyzed by SEM (Figure 5). OSA-starch particles were spherical or irregular, like deflated balloons, with a smooth surface and a diameter of 1–10 µm (Figure 5a). Nano spray-dried chitosan particles were spherical, some of them smooth and the others rough, with a diameter of 0.5–2 µm (Figure 5b). Encapsulation with this technique seems to produce exclusively spherical structures [43].

As the amount of nano spray-dried particles was insufficient to carry out planned studies, regular spray-drying instrumentation has been applied. The chitosan particles were obtained from the emulsion composed of chitosan/oil/Tween 80 at 1/0.2/0.1. The size of the particle was between 1 to 7 µm (Figure 5c), meaning that they were larger compared to the nano spray-dried chitosan ones.

- By freeze-drying

Particles obtained by freeze-drying were also analyzed by SEM (Figure 5). Blocks of OSA-starch can be observed with air bubbles inside (Figure 5d). Chitosan emulsions dried in this process looked like flakes (visual observation), whose surface is covered with round protuberances (Figure 5e).

### 2.2. Synthesis of THC-Loaded Polysaccharide Particles

#### 2.2.1. Characterization of THC

^1^H-NMR analysis was performed to verify if no undesirable signals were present. ^1^H-NMR (DMSO-d_6_, 400 MHz) of 1,7-bis(4-hydroxy-3-methoxyphenyl)heptan-3,5-dione: δ 15.58 (s, 0.4H, H enol); 8.78 (d, 2H, 8.2 Hz, h + h’); 6.76 (dd, 2H, 1.8/13.7 Hz, f); 6.65 (dd, 2H, 2.5/8.0 Hz, d); 6.56 (m, 2H, 1.9/8.0 Hz, e); 5.74 (s, 0.5H, a enol); 3.73 (s, 6H, g + g’); 3.69 (s, 1H, a diketone); 2.74 (m, 4H, b + b’); 2.66 (m, 2H, c or c’); 2.56 (m, 2H, c or c’). The signals were those characteristic of THC and the spectrum was similar to the one obtained by Trivedi et al. [44]. This technique can be used to determine the ratio between the diketone and the keto-enol form, thanks to the signals of proton of the diketone and the signals of the enolic proton. It was equal to approximately 1:1 in DMSO, as the integration of the two protons of the diketone in-between the two carbonyl groups, “a diketone,” was equal to 1 and the integrations of the enolic proton and proton, “a enol,” were, respectively, equal to 0.4 and 0.5.

**Figure 6:**
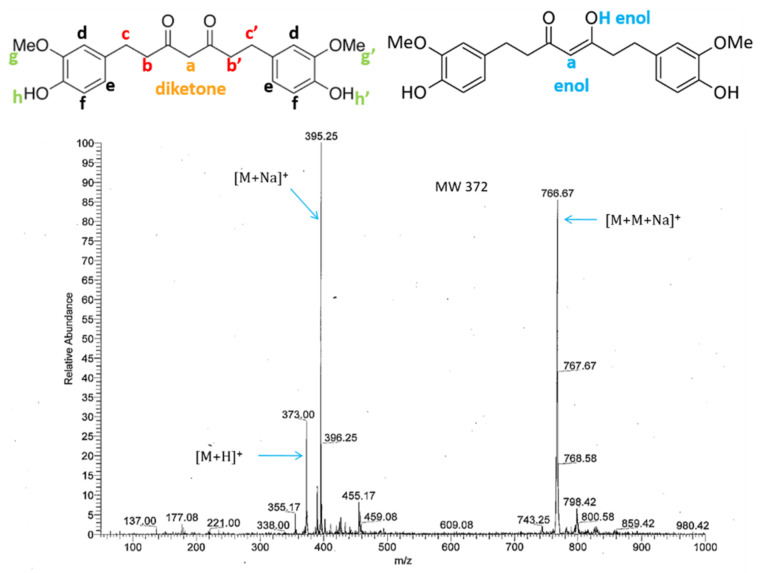
Direct infusion ESI(+)-mass spectrum of commercial THC with labeled protons.

The mass spectrum of THC was recorded using positive electrospray ionization (ESI) by direct infusion (Figure 6). Several ions can be observed at *m*/*z* 373, 395 and 767, corresponding to the hydrogenated molecule [M + H]^+^, the cationized molecule [M + Na]^+^ and a cationized dimer [M + M + Na]^+^, respectively.

#### 2.2.2. Anti-TCTB Properties of THC

The addition of 10 µM of THC alone directly onto the solid medium have not shown any inhibition of the fungal growth (data not shown). Consequently, the effect of THC on the production of toxins could be evaluated because this product does not interfere with growth at this concentration.

**Figure 7:**
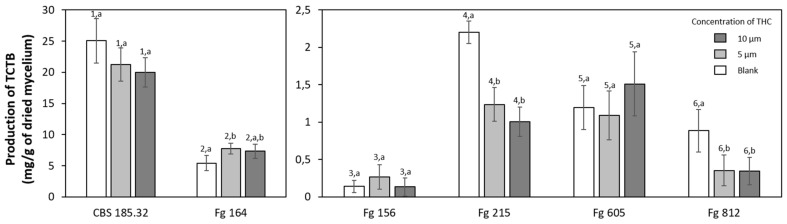
Production of TCTB(s) for six different strains, CBS 185.32, Fg 164, Fg 156, Fg 215, Fg 605 and Fg 812, of *F. graminearum* in liquid medium amended by (white) blank; (light grey) THC 5 µM; (grey) THC 10 µM after 14 days at 25 °C and 70% R.H. Error bars correspond to the standard deviation of measurements of five Petri dishes. Different letters indicate significant differences (*p* < 0.05) within an experiment (marked with figures).

As is well known, *F. graminearum* produces trichothecenes B (TCTBs). The accumulation of toxins has been observed for six different strains of *F. graminearum* (Figure 7), which all produce the same kind of TCTBs, namely DON and 15-acetyl-DON. In blanks, the amount of TCTBs varied from 0.14 to 25 mg/g of dried mycelium for Fg 156 and CBS 185.32, respectively. The accumulation of TCTB by Fg 215 and Fg 812 was, indeed, significantly reduced, up to 54% for 10 µM of THC on Fg 215. For the strain CBS 185.32, the reduction was not significant. With regard to the three other strains, the results were ambiguous. No trend was observed for Fg 156 and Fg 605, even if changes were not significant. Finally, an increase in the mycotoxigenesis was noted for Fg 164. For the rest of the study, microbiological tests were performed on the model strain CBS 185.32, as it is a model strain producing the largest amount of toxins.

#### 2.2.3. THC-Loaded Particles

THC (5% *w*/*w*) was added directly into rapeseed oil before mixing the two phases, i.e., aqueous and oily. Encapsulating THC in oil droplets did not drastically change their size.

**Figure 8:**
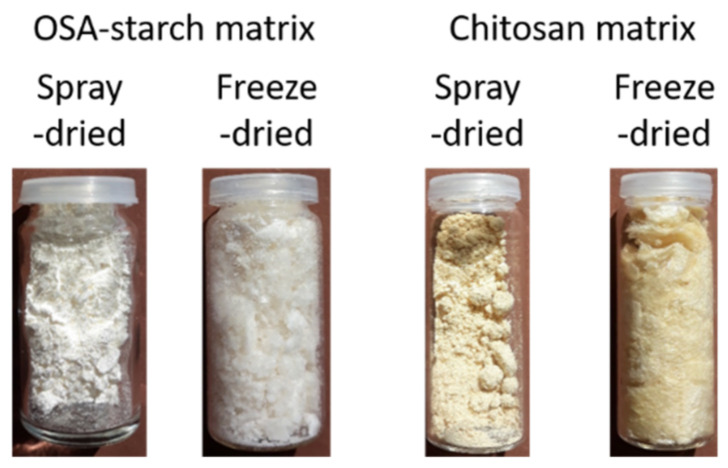
Pictures of THC-loaded particles.

The drying protocol was the same as for the blank particles. It is important to recall that spray-dried starch particles have been obtained with the nano spray-dryer and chitosan ones with the conventional spray-dryer. Particles have different physical appearances (Figure 8), some of them being powder-like (thin or coarse) or more like flakes. Their aspect did not differ from non-loaded particles, except for spray-dried chitosan THC-loaded particles, which are more yellow-orange, than non-loaded ones, which are yellowish.

**Figure 9:**
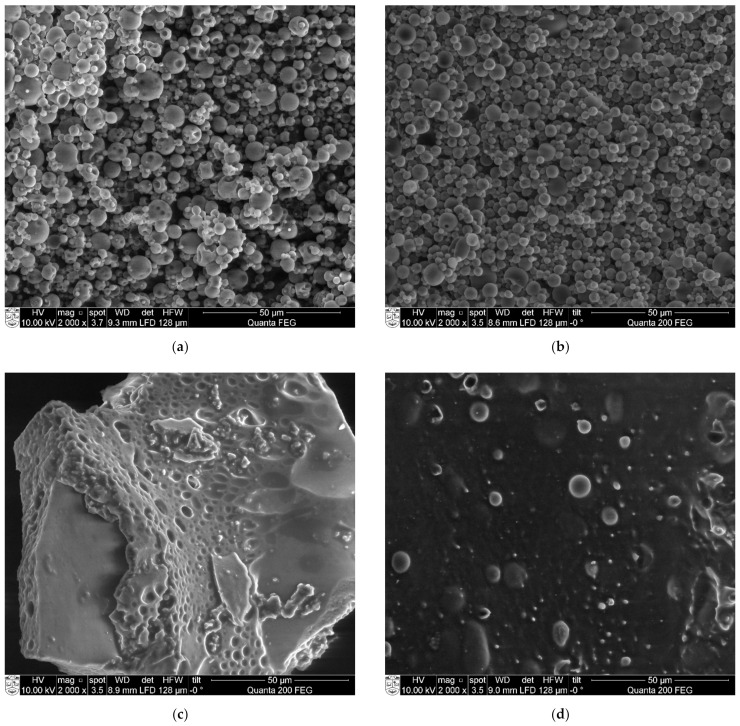
SEM views (2000×) of THC-loaded particles: (**a**) nano spray-dried OSA-starch; (**b**) spray-dried chitosan; (**c**) freeze-dried OSA-starch; (**d**) freeze-dried chitosan.

No crystals of THC could be observed on the microscopy views of THC-loaded particles (Figure 9).

### 2.3. Properties of THC-Loaded Particles

#### 2.3.1. Release Study

**Figure 10:**
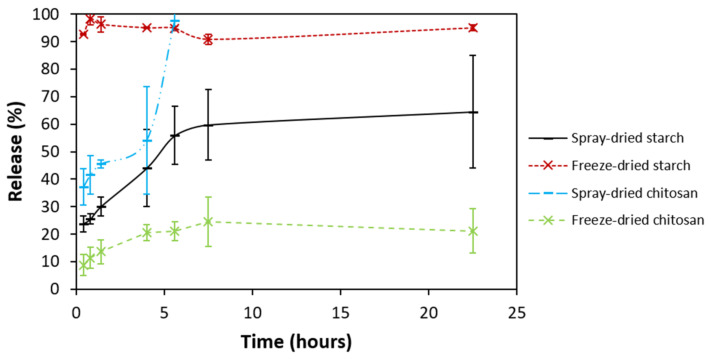
Release of THC from THC-loaded particles in ethanol at ambient temperature. Error bars correspond to the standard deviation of measurements performed in triplicate.

The release studies of THC from different polysaccharide matrices were carried out in ethanol. The results are shown in Figure 10. The efficient release of THC from spray-dried starch particles was observed, with the whole THC content being released into ethanol directly after the dispersion of particles. A gradual release of THC was noticed for other matrices, with the plateau being reached after 6–7 h.

#### 2.3.2. Thermal Analysis

**Figure 11:**
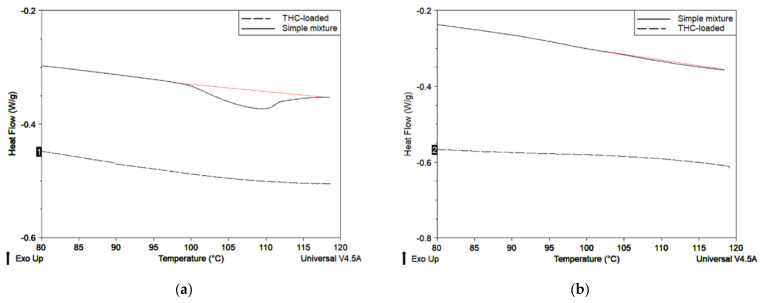

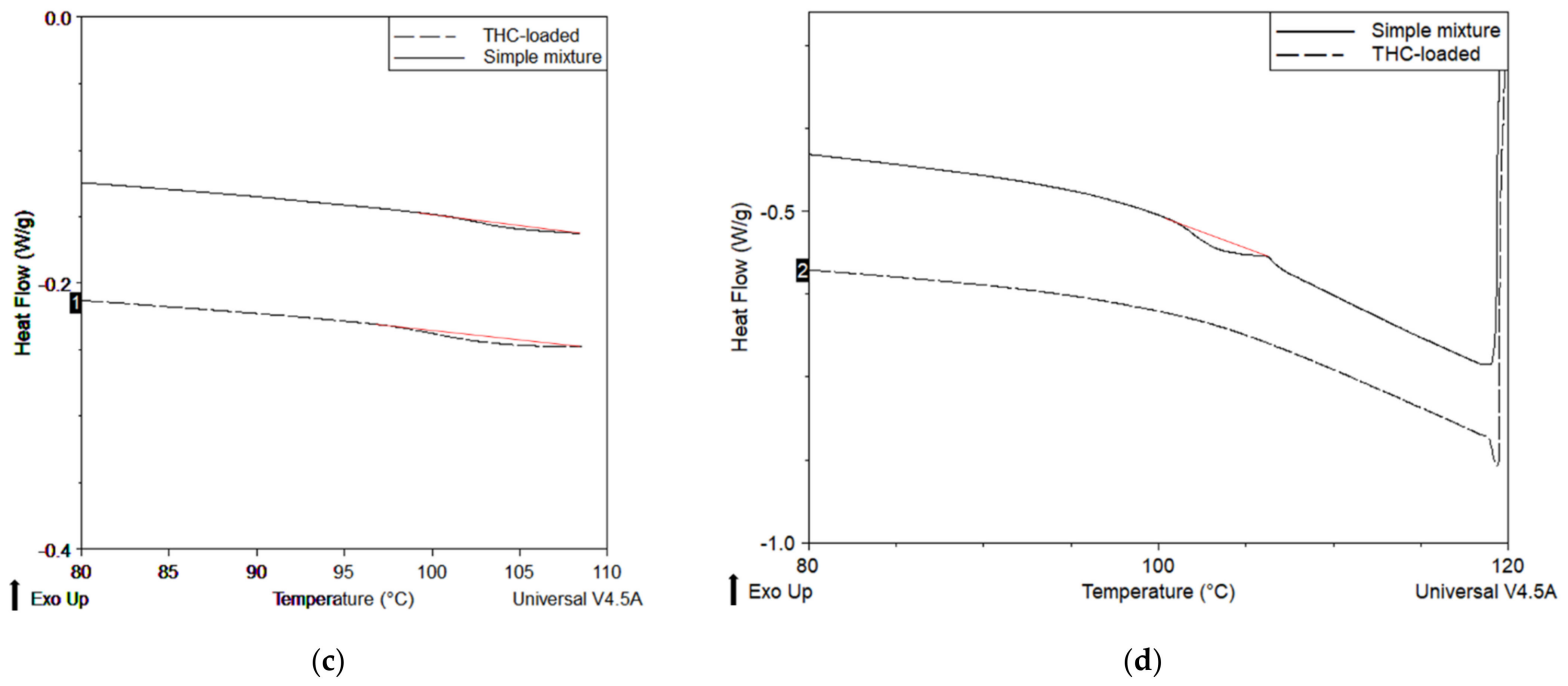
DSC thermograms of THC-loaded particles and simple mixtures of non-loaded particles and THC, for (**a**) nano spray-dried OSA-starch; (**b**) spray-dried chitosan; (**c**) freeze-dried OSA-starch; (**d**) freeze-dried chitosan.

The THC melting peak was found at 100 °C (data not shown). In all physical mixtures of THC and control particles, the melting of THC can be observed (Figure 11, solid lines). On the opposite, in almost all THC-loaded particles, there was no THC melting peak (Figure 11a,b,d, dotted lines). This could indicate that THC is either protected inside the particles or in an amorphous form. Freeze-dried THC-loaded starch particles are the exception, since the melting peak is visible (Figure 11c). This could be due to the fact that THC was not protected in those particles.

#### 2.3.3. Radical Scavenging Activity

THC has a higher ability to scavenge radicals than BHT, which is commonly used as antioxidant in the industry. The DPPH inhibitions of THC and BHT were 73% and 43%, respectively.

Control particles did not present a real scavenging activity, with only up to 4% of inhibition of the DPPH radical (Table 1). On the other hand, almost all THC-loaded particles quenched the DPPH radical, except the THC-loaded spray-dried starch particles.

THC-loaded freeze-dried starch particles inhibit 80% of the radical, which is close to what was found with pure THC.

#### 2.3.4. Antifungal Activity against *F. graminearum*

- With OSA-Starch

As seen on Table 2, the fungal growth was reduced by using these newly synthetized particles. Starch particles, free of THC, inhibited the fungal development. THC loading improved the efficiency of freeze-dried and spray-dried particles by 14% and 5%, respectively. As a comparison, THC alone in oil but twice as concentrated gave an 11% increase compared to the rapeseed oil.

- With chitosan

Chitosan particles demonstrated a much higher antifungal activity than starch ones. Indeed, a concentration of 0.4 g/L of THC-loaded chitosan particles gave the same fungal inhibition at 4 days as a concentration of 13.3 g/L of starch particles (curves not shown). For clarity purposes, the antifungal activity of chitosan particles was evaluated by calculating the concentration at which 50% of the growth is reduced (IC_50_) (Table 3). THC loading improved the activity of freeze-dried particles but had no effect on spray-dried particles. Spray-dried particles were the most efficient against *F. graminearum*, with the lowest IC_50_ at 0.5 ± 0.2 g/L.

#### 2.3.5. Anti-TCTB Activity

**Figure 12:**
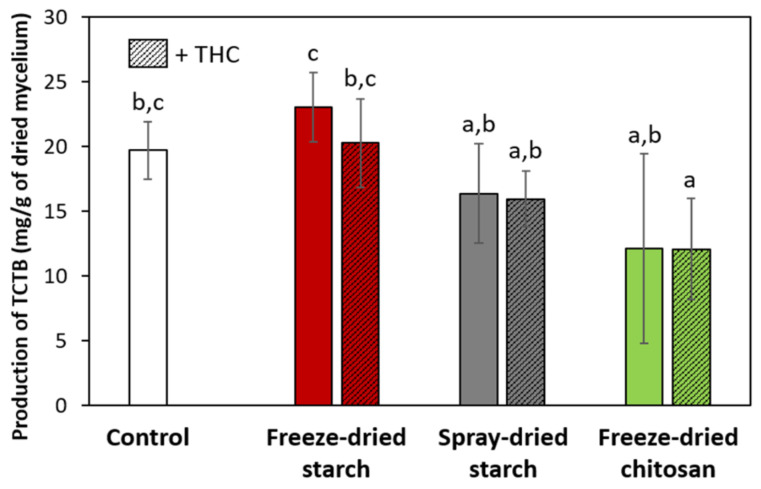
Production of TCTBs by *F. graminearum* CBS 185.32 in liquid medium amended by non-loaded or THC-loaded particles at 125 mg/L after 14 days of incubation at 25 °C and 70% of relative humidity. Error bars correspond to the standard deviation of measurements of 5 Petri dishes. Values with different letters presented significant differences.

The data on the production of TCTB in the presence of particles, with or without THC, are given in Figure 12. For both conditions with spray-dried chitosan particles, the mycelium did not grow and the evaluation of the toxin production was, therefore, impossible.

Except for THC-loaded freeze-dried chitosan particles, with 39% of inhibition, no significant reduction of the TCTB accumulation can be noticed.

The production of toxins was lightly activated by freeze-dried OSA-starch particles, maybe because they act as a carbon source. The addition of THC counterbalanced this undesirable trend. The difference between these two conditions is about 10%, which is what was determined previously for THC (see Figure 7). It strengthens the hypothesis that THC is fully released from those starch particles, as demonstrated by the release study, DSC and DPPH test.

## 3. Discussion

### 3.1. Preparation of Particles

The drying process of the emulsions seems to determine the shape of the synthesized particles. The spray-dried particles obtained in this work were spherical, whatever the biopolymer matrix used. For comparison, chitosan nanoparticles obtained by Ngan et al. [45] were also spherical. Structures that look like deflated balloons have been observed with different wall materials, such as cyclodextrin [46], OSA-starch [47], maltodextrin or gum Arabic [48,49]. As far as freeze-dried particles are concerned, various shapes have been described, which are attributed to the different shapes of water crystals before water sublimation in the lyophilization process. The higher the dry weight in the solution, the denser the particles. The chitosan freeze-dried particles obtained in this study seemed to be thin, as the dry mass of the emulsions is low (1.3%). Sponge-like structures have been described by Croisier and Jérôme [50] on freeze-dried chitosan and poly(ethylenimine) (1% < dry mass < 10%) by Khan et al. [51]. Moreover, even denser structures were observed with the starch particles synthetized in this work (dry mass 36%).

### 3.2. Physicochemical Characterization of THC-Loaded Particles

The four kinds of synthetized particles exhibited different behaviors. The freeze-dried starch particles loaded the most THC, but did not protect it. THC was, indeed, rapidly released into ethanol and could easily react with DPPH radicals in methanol. Moreover, THC was available in its crystal form according to DSC. The THC loading of these particles also gave the best improvement of antifungal and anti-TCTB activities, compared to other THC-loaded/non-loaded particles. All these facts tend to show that, in this particular system, THC is poorly encapsulated, but in a significant amount. Ballesteros et al. [48] also found that the freeze-drying process tends to encapsulate more phenolic compounds than spray-drying.

Both starch and chitosan spray-dried particles seemed to better protect THC and to extend its time of release. It took one day to reach the plateau with those particles. Hu et al. [42] also reached a plateau of release for curcumin from spray-dried OSA-starch particles after one day. Curcumin was released faster, i.e., in two hours from chitosan spray-dried particles in other studies [38,40]. The good protection could also explain why the THC melting peak is not present in THC-loaded spray-dried particles. Regarding the DPPH test, the small inhibition value obtained for chitosan particles can be due to a slow release of THC in methanol. A small inhibition for spray-dried starch particles would have also been expected, as a low part of the phenolic compound should be released after 25 min, which is the duration of the DPPH tests. The discrepancy in the THC amount and availability over time may be due to the release itself. The starch freeze-dried matrix may be looser and/or easier to break, causing a faster THC diffusion, compared to the spray-dried starch 3D network. It could be possible that the way water is evaporated as speed, stress, etc. in each technique may play an important role on the density and tightness of the network, thus influencing the release rate. For example, Cho et al. [52] suggested that the drug release rate from chitosan/tripolyphosphate microspheres is influenced by the density of their matrix. A more rigid network and a slower release rate was associated with the higher content of the crosslinking agent.

Finally, for freeze-dried chitosan particles, their low efficiency must come from a low THC loading amount as the number of hydrophobic groups available for oily phase solubilization in the chitosan structure is much lower when compared to OSA-starch.

### 3.3. Activity of THC and THC-Loaded Particles against F. graminearum

#### 3.3.1. Effect of THC, OSA-Starch and Chitosan Alone

THC did not have any effect on the growth of *F. graminearum* at the studied concentration of 10 µM, probably because its concentration was too low. Therefore, the activity of THC against the production of toxins could been assessed at two concentrations of THC.

The reduction of the mycotoxigenesis by phenolic compounds, such as chlorogenic or caffeic acid [53], resveratrol [54] or *p*-hydroxybenzoic acid [55], have been reported in the literature. For instance, caffeic acid reduced the amount of TCTBs of *F. graminearum* CBS 185.32 by 70% and 20% after 8 and 21 days of incubation, respectively. Such an inhibition is component-dependent. For example, in the study of Ferruz et al. [56], all the tested phenolic compounds, except *p*-coumaric acid, reduced the amount of toxins produced by *F. langsethiae* or *F. sporotrichioides*. The impact also depends on fungal species, as phenolic compounds can reduce the production of mycotoxins by *F. graminearum* and *F. langsethiae*; however, by *F. poae* treated with ferulic acid, there was no clear trend [57].

Several studies have demonstrated that the effect of active molecules is strain-dependent [53,58,59]. Therefore, we have studied the effect on the accumulation of mycotoxins by six different strains of *F. graminearum*, named CBS 185.32, Fg 164, Fg 156, Fg 215, Fg 605 and Fg 812, knowing that numerous strains are present on fields. No specific trend can be observed, but the activity of THC is certainly strain-dependent. In the literature, various strains seemed, indeed, to react differently. In a work of Ponts et al. [55], caffeic and syringic acids inhibited the production for one strain of *F. graminearum* and activated another one. In another work on two *Fusaria*, the most sensitive strain for *F. culmorum* was the one producing the highest amount of toxins, and for *F. graminearum*, the two strains producing the lowest amount of toxins were the most sensitive [60].

The efficiency of phenolic compounds can sometimes be related to their antioxidant properties. Indeed, many authors found a correlation between antioxidant and antimycotoxigenic properties [61,62] and some other did not find any correlation [8,63]. As far as *F. graminearum* is concerned, the production of TCTBs is linked with the fungal stress-response [64]. In our study, THC possessed both radical scavenging and anti-TCTBs activities, which could be related. However, deeper studies should be performed to determine whether antioxidant properties are responsible of the mycotoxin-reducing activity of THC. Thus far, THC has shown good radical scavenging activity of the DPPH radical, almost twice as high than the one of BHT. According to Portes et al. [9], THC was more antioxidant than curcumin, eugenol or BHT.

OSA-starch as well as chitosan particles without THC exhibited antifungal properties. This could be due to electrostatic interactions between fungal membrane and negatively charged octenyl residues of OSA-starch in one case or protonated ammonium groups of chitosan in the other case. The antifungal activity of chitosan is well-known and already widely described in the literature [65,66,67,68]. Chitosan nanoparticles were found to be more efficient than non-processed chitosan dissolved in solution against *F. graminearum* [69] and *Staphylococcus aureus* [45]. Regarding antioxidant properties, chitosan particles possessed a small antioxidant capacity. However, this antioxidant activity has been controverted in the literature. It is often said to be linked with its chelating activity [70]. Intrinsic antioxidant activity of chitosan could eventually come from an unshared pair of electrons on nitrogen atoms, if they are not protonated (3% of amine groups were indeed not protonated, unpublished results). This could be the case, since, in the emulsions from which particles are made, chitosan is soluble in its protonated form. The small antioxidant capacity of freeze-dried chitosan particles could explain its activity against mycotoxins.

#### 3.3.2. Effect of THC-Loaded Particles

The addition of THC into particles increased the apparent THC concentration in the medium, thus making possible the observation of its antifungal properties. The antifungal activities of phenolic compounds are often associated with their lipophilic properties, which are responsible for their ability to interfere with fungal membrane. Changes in the membrane permeability or fluidity and apparition of pores in membranes, thus causing leakage of intracellular content, are often reported [62,71,72,73].

Many examples showing an increased efficiency of the trapped compound compared to the free one can be found in the literature. Chitosan/lecithin particles, loaded with kaempferol, have inhibited the growth of *Fusarium oxysporum* by 67% after 60 days of culture, whereas free kaempferol was no longer efficient after 20 days [74]. Amoxillicin-loaded spray-dried chitosan nanoparticles were more efficient than free amoxillicin, with a minimum inhibitory concentration six times lower for particles [45]. The release of the herbicide 3-hydroxy-5-methylisoxazole was delayed up to 160 h thanks to the encapsulation in chitosan through a microencapsulation by an emulsion process [75]. In another study, carbendazim, a synthetic fungicide, was used in composite films of chitosan. In total, 76% of carbendazim was released after 48 h from the composite. A seedling oilseed rape stem was 100% protected from *Sclerotinia sclerotiorum* (Lib.) de Bary until 21 days, while the fungicide alone was efficient the first week only [61].

In this study, the efficiency of THC was indeed increased, but mainly due to a higher amount in the medium. The efficiency of chitosan particles seemed to come from the polysaccharide matrix itself.

## 4. Materials and Methods

### 4.1. Material

Tetrahydrocurcumin (THC) was purchased from SABINSA EUROPE GmbH. Butylated hydroxytoluene (BHT, Acros, Fisher Scientific, Waltham, MA, USA), 2,2-diphenyl-1-picrylhydrazyl (DPPH, Sigma-Aldrich, Saint-Quentin-Fallavier, France), glacial acetic acid (AA) (≥99%), potassium bromide and solvents used were of analytical grade.

Chitosan, with an $\bar{M_{n}}$ of 78 kg/mol and a deacetylation degree of 92%, was purchased form SPN Agrobio^®^ (Varades, France). An OSA-starch octenylsuccinic acid modified waxy maize starch was used. Rapeseed oil was from UAB “Rokiškio aliejinė,” Lithuania. Tween 80 was purchased from Sigma.

Potato Dextrose Agar (PDA) medium (potato starch 4 g/L, dextrose 20 g/L, agar 15 g/L) was provided by Biokar. Carboxymethylcellulose (CMC) was provided by Sigma-Aldrich. All salts used in the media were of analytical grade.

TCTB standard solutions were provided by Romer Labs (Baulkham Hills, Austria).

The following strains of *Fusarium graminearum* were used during this study: Fg 156, Fg 164, Fg 215, CBS 185.32, Fg 605 and Fg 812. All strains present DON/15-acetyl-DON chemotype and belong to INRA-MycSA’s laboratory collection (Villenave d’Ornon, France). INRA-MycSA’s strains are deposited in the International Center for Microbial Resources—Filamentous Fungi [76].

### 4.2. Methods

#### 4.2.1. Preparation of Spray-Dried and Freeze-Dried Particles

OSA-starch was weighted taking into the account the moisture content to form aqueous solutions of 20% (*w*/*w*) or 30% (*w*/*w*). The moisture content was determined with a drying balance (Kern MRS 120-3). Distilled water was added and the solution was heated up to 70 °C until the total dissolution of starch. After cooling, the solution was light yellowish. Water was added to the solution to compensate losses due to water evaporation. Solutions were stored in the fridge for a maximum of one week.

If needed, THC (5%, *w*/*v*) was suspended in rapeseed oil at ambient temperature under magnetic stirring at 400 rpm for two days.

OSA-starch (20 g, 18 g and 16 g) aqueous solution and oil (0 g, 2 g and 4 g) were poured in a narrow beaker, where they were homogenized with IKA^®^ T25 digital ULTRA-TURRAX for 5 to 20 min, at ambient temperature, at 10,000 rpm or 13,000 rpm. Emulsions without THC added in the oil were produced as control. Both THC-loaded and control OSA-starch particles were produced from the emulsions containing 16 g of OSA-starch solution at 20% and 4 g of oil solution.

An aqueous solution of chitosan-AA was prepared using chitosan at 10 g/L and acetic acid (AA) at 10 mL/L. Distilled water was added very progressively. The mixture was left overnight under stirring until total dissolution was achieved.

Chitosan–AA aqueous solution (500 g), oil (1 g) and Tween 80 (500 mg) were poured in a narrow beaker, where they were homogenized with IKA^®^ T25 digital ULTRA-TURRAX for 20 min, at ambient temperature, at 10,000 rpm. Emulsions without THC added in the oil were produced as control. The corresponding ratio (w) Chitosan/oil/Tween 80 was 1/0.2/0.1.

Two types of spray-drying techniques were employed to obtain dry polysaccharide particles, i.e., nano spray-drying and spray-drying.

The prepared modified starch and chitosan emulsions were diluted 10 to 20 times and spray-dried using a nano spray-dryer (Büchi Nano Spray Dryer B-90, Songjiang District, Shanghai). The air flow was 110 L/min, the inlet temperature was 100 °C, the outlet temperature 45 °C and the nozzle size was 7 µm.

Spray-drying of chitosan emulsions was only achieved by using the LabPlant SD-06 spray-dryer (Keison, Chelmsford, Essex, UK). The inlet temperature was 170 °C, the outlet temperature 70 °C and the air flow was 200 m^3^/h.

Using the freeze-drying technique, emulsions were frozen in the freezer overnight and then dried using SP VirTis Bench Top 2K freeze-dryer.

#### 4.2.2. Physicochemical Characterization

- Dynamic Light Scattering

A Delsa^TM^ Nano C particle size analyzer (Beckman Coulter) was used to determine the size of the droplets and polydispersity index of starch and chitosan-based emulsions according to the cumulative distribution of intensity. The samples were measured in triplicate with 20 scans each and averaged. The Delsa Nano C uses photon correlation spectroscopy, which determines particle size by measuring the rate of fluctuations of the laser light intensity scattered by particles. The non-negative least-squares (NNLS) algorithm was used to analyze dynamic light scattering data for the droplet size distribution.

- Differential Scanning Calorimetry

DSC was employed to study the behaviour of THC around its fusion temperature. It was performed with TA Instrument Q100 RCS. Approximately 4 mg of compound were placed in an aluminum pan and heated from 20 °C to 110 °C with a heating rate of 10 °C/min under nitrogen (25 mL/min). A first cycle with a plateau at 90 °C was necessary to eliminate water. THC-loaded particles were compared with a physical mixture of the control particles and THC. The amount of THC in physical mixture was the same as in the THC-loaded particle.

- Scanning Electron Microscopy

SEM observations of obtained starch and chitosan particles were performed on an FEI Quanta 200 FEG scanning electron microscope. Stubs with double-face tape were used as supports for a thin layer of powdered sample.

- Mass spectrometry

The analysis was performed on a linear trap quadrupole (LTQ) mass spectrometer (Thermo Fisher Scientific) in positive ion mode using direct infusion of THC in methanol (0.1 mg/mL). ESI source parameters were as follows: capillary voltage +20 V; tube lens voltage +90 V; capillary temperature 300 °C; Sheath and Auxiliary Gas flow (N_2_), 8 and 5; Sweep gas 0; Spray voltage 3.6. MS spectra were acquired by full range acquisition covering *m/z* 50–1000.

- ^1^H-Nuclear Magnetic Resonance spectroscopy

^1^H-NMR spectrum of THC was obtained using a Liquid-state 400 MHz NMR spectrometer (Bruker AVANCE I) in deuterated dimethylsulfoxide (DMSO) with 16 scans.

- Release study

THC-loaded or non-loaded particles (10 mg) and ethanol (10 mL) were put in vials under stirring. Samples (500 µL) were taken and replaced by ethanol (500 µL). Samples were rapidly analyzed by UV-spectrophotometry at 280 nm. The absorbance of THC was determined by subtracting the absorbance of blank particles. The concentration of THC was then calculated thanks to a calibration curve and a correction factor was added to take into account the dilution of each sample. The release percentage was calculated by taking into account the THC amount added to the emulsions at the first step of the synthesis of the particles. The studies were performed in triplicate.

- Radical Scavenging Activity

The DPPH technique was performed on THC, BHT and THC-loaded particles. The radical solution was freshly prepared and stored in the fridge when not used. DPPH (1.97 mg) was added in a 50 mL graduated flask. After adding methanol, the solution at 10^−4^ M was poured into an amber bottle. Solutions of THC or BHT at 10^−3^ M and solution of THC-loaded particles at 12.4 g/L were also prepared in methanol. A total of 5 mL of the DPPH solution and 150 µL of reactant solutions were reacted in amber vials under stirring for 25 min. Centrifugation at 3000 rpm for 3 min was needed for particle samples before analysis. 500 µL of homogeneous reaction solution were quickly poured into a quartz cell and analyzed using the spectrophotometer in the wavelength range from 250 to 600 nm. The control solution followed the same preparation, but 150 µL of methanol were added instead of the reactant solutions. The value of the absorbance intensity at 517 nm was compared to the one of the control solution and the inhibition percentage was calculated with Equation (1), where A is the absorbance intensity at 517 nm:

(1) $$\%Inhibition=\frac{A\left(control\right)-A\left(antiox.\;molecule\right)}{A\left(control\right)}\times 100$$

#### 4.2.3. Microbiological Tests

Four agar cubes were cut from a Petri dish of newly grown mycelium. They were incubated in 20 mL of CMC medium (CMC 15 g/L, yeast extract 1 g/L, MgSO_4_ 7H_2_O 0.5 g/L, NH_4_NO_3_ 1 g/L, KH_2_PO_4_ 1 g/L) and put under agitation (180 rpm), in the dark and at 25 °C. After 4 days of growth, the suspension of spores was filtrated with a 100 µm filter. The spores were counted with a Malassez cell on a microscope X40 (Leica) and the desired concentration of the solution of spores was adjusted with distilled water.

The mycelial growth inhibition test was performed on Petri dish of 85 mm in diameter filled with 15 mL of PDA. The medium was supplemented with tested molecules, which had been mixed when the medium was hot and liquid. The control condition corresponded to the PDA medium. Approximately 10 µL of the solution of spores, containing 100 spores, were inoculated on the center of the dish. Petri dishes were incubated at 25 °C at 70% of relative humidity in the dark. Two perpendicular diameters were measured every day. All conditions were performed in triplicate.

The percentages of inhibition at day 4 were expressed as shown in Equation (2):

(2) $$\%Inh=\frac{Average\;diameter\;of\;blank-Average\;diameter\;of\;condition\;i\;\;}{Average\;diameter\;of\;blank}\times 100,$$

The determination of the IC_50_ required that the product has been evaluated at several concentrations. Then, at day 4, the percentages of inhibition were plotted versus the logarithm of the concentrations of the tested product. A linear regression was performed. The IC_50_ was calculated according to Equation (3).:

(3) $$IC_{50}=10^{\frac{50-b}{a}},$$

where b is the intersection at the origin of the regression line and a is the slope of the regression line.

Strains were grown in Minimal Synthetic liquid medium (glucose 20 g/L, KH_2_PO_4_ 0.5 g/L, K_2_HPO_4_ 0.6 g/L, MgSO_4_ 17 mg/L, (NH_4_)_2_ SO_4_ 1 g/L, traces element of Vogel 10 µg/mL). The Minimal Synthetic solution (8 mL) was poured in small Petri dishes (diameter = 55 mm) and inoculated with 2 × 10^5^ spores/mL of the solution of spores. Petri dishes were incubated in the dark at 25 °C at 70% relative humidity. After 15 days, fungal liquid cultures were transferred into plastic tubes weighted beforehand and then centrifuged at 3360× *g* for 5 min. Supernatants were poured into new tubes and stocked at −20 °C until analyses. Mycelia were placed at −80 °C and then lyophilized during 48 h. The mass of dry biomass was determined by weighing.

TCTBs were extracted by a liquid-liquid extraction with aqueous medium (4 mL) and ethyl acetate (8 mL). The organic upper phase (5 mL) was then transferred into a new glass tube and evaporated under nitrogen flux. The different dry extracts were resolubilized in 400 µL of methanol/water (1:1). The extracted samples were filtrated through a 0.22 µm filter and then run on an Agilent Technology 1100 liquid chromatography system as already described by Gauthier et al. and Montibus et al. [53,63].

All experiments were done with at least three repetitions. Data for TCTB production and fungal growth were reported as mean values ± standard deviation of three to five replications. Mean values were compared at the 5% significance level using Conover-Iman test (control vs. treated) with the XLSTAT software.

## 5. Conclusions

In this study, THC was encapsulated in polysaccharide matrices for biofungicide applications. THC-loaded rapeseed oil and either OSA-starch or chitosan/AA/Tween 80 solutions were employed in the preparation of oil in water emulsions with nanosized droplets. Spherical and sheet-like structures were obtained by using spray-drying and freeze-drying techniques, respectively. Except freeze-dried OSA-matrices, THC melting peak was not visible in DSC curves. Furthermore, it was demonstrated that THC can be gradually released from the particles. THC-loaded particles have shown promising antifungal properties.

For the targeted antifungal application, spray-dried chitosan particles were the most promising systems. The properties of THC-loaded OSA-starch particles potentially could be exploited in other applications such as food preservation, as the excellent radical scavenger properties of DPPH were revealed.

## Figures and Tables

**Table 1:** Inhibition percentage (%) of the DPPH radical at 517 nm. Values are expressed as mean value ± standard deviation of duplicate.

Polymer Matrix	Control Particles	THC-Loaded Particles
Spray-dried OSA-starch	2	0
Freeze-dried OSA-starch	0	80 ± 2
Spray-dried chitosan	2	12 ± 4
Freeze-dried chitosan	4	13 ± 2

**Table 2:** Inhibition percentage of the growth of *F. graminearum* CBS 185.32 on PDA medium supplemented with OSA-starch particles or rapeseed oil at 13.3 g/L, with and without THC, after 4 days of inoculation at 25 °C and 70% of relative humidity. Values are expressed as the mean value ± the standard deviation of the triplicate. Asterisks indicate significant differences (*p* < 0.05) compared to the control compound.

	Control Compound	THC-Loaded Compound
Freeze-dried OSA-starch	7 ± 1	21 ± 2 *
Spray-dried OSA-starch	12 ± 1	17 ± 1 *
Rapeseed oil	2 ± 1	13 ± 3 *

**Table 3:** IC_50_ of *F. graminearum* CBS 185.32 of freeze-dried or spray-dried chitosan particles after 4 days of inoculation at 25 °C and 70% of relative humidity. Measurements were performed in triplicate.

	Control Particles	THC-Loaded Particles
Freeze-dried chitosan	0.9 ± 0.1	0.65 ± 0.02
Spray-dried chitosan	0.5 ± 0.2	0.6 ± 0.3

## Data Availability

Not applicable.

## Sample Availability

Samples of the compounds are available from the authors.

## Abbreviations

AA	Acetic acid
BHT	Butylated hydroxytoluene
DPPH	2,2-diphenyl-1-picrylhydrazyl (radical)
OSA	Octenylsuccinic acid
TCTB	Trichothecene B
THC	Tetrahydrocurcumin
